# QuantiFERON-TB Gold In-tube test for the diagnosis of active and latent tuberculosis in selected health facilities of Addis Ababa, Ethiopia

**DOI:** 10.1186/s13104-018-3410-x

**Published:** 2018-05-11

**Authors:** Selam Niguse, Kassu Desta, Gebremdihin Gebremichael, Atsebeha Gebrezgeaxier, Mulluwork Getahun, Desta Kassa

**Affiliations:** 1 Medical Microbiology and Immunology Department, Mekelle University, PO. Box: 1871, Mekelle, Ethiopia; 2 Department of Medical Laboratory Science, Addis Ababa University, Addis Ababa, Ethiopia; 3 HIV/AIDS and TB Research Directorate, Ethiopian Public Health Institute, Addis Ababa, Ethiopia

**Keywords:** Latent tuberculosis, Active tuberculosis, QuantiFERON-TB IN-Gold, Tuberculin skin test, Human immunodeficiency virus, Addis Ababa

## Abstract

**Objective:**

To determine the performance of QuantiFERON-TB IN-Gold for the diagnosis active tuberculosis and latent tuberculosis.

**Results:**

A total of 213 participants (136 tuberculosis suspects, 66 latently infected) were enrolled. Of 213, 21 (15.4%) of the tuberculosis suspects and 3 (4.5%) of the latent tuberculosis groups were human immunodeficiency virus infected. The sensitivity, specificity, positive and negative predictive value of QuantiFERON-TB IN-Gold for the diagnosis of active tuberculosis was 70.3% (26/37), 49.5% (49/99), 34.7% (26/75) and 83.1% (49/59) respectively. A kappa value of 0.316 (p = 0.001, 95% CI 1.605–1.609) between QuantiFERON-TB IN-Gold and tuberculin skin test were found.

## Introduction

Tuberculosis (TB) is still one of the deadly infectious diseases worldwide. It is estimated that around one-third of the world’s population is infected with latent TB (LTBI) with 5–10% life time risk of developing active TB [1, 2]. Presence of good diagnosis is important for the control and ultimate elimination of the disease [3]. However, diagnosis of LTBI and active TB using the existing tools is challenged by low sensitivity (in smear microscopy); need of prolonged time for results and need of sophisticated laboratories and expertise (in TB culture) [4]; relatively expensive and heterogeneous diagnostic accuracy (in molecular techniques) [5]; false positive reactions [6], lower sensitivity in immune-suppressed peoples [6, 7], anamnestic recall of immunity [8], potential for inter- and intra- operator variability of results [9], inconvenience for patients in tuberculin skin test, a test, based on the fact that infection with *M. tuberculosis* bacterium produces a delayed-type hypersensitivity skin reaction to certain components of the bacterium [10]. Putting all the challenges of the techniques together reinforces the need for improved diagnostic tools. Of the different tests developed, in the last 2 decades, interferon gamma release assays (IGRAs) has been introduced as an alternative test for the diagnosis of active and latent TB. There are 2 forms of IGRAs, the quantiFERON and the T-SPOT.TB. The QuantiFERON-TB Gold In-tube (QFT-GIT) uses enzyme-linked immunosorbent assay (ELISA) to measure antigen-specific production of IFN-γ [11]. The performance of the QFT-GIT has been evaluated either for active or latent TB, in different settings and showing a promising results. However, there is a scarcity of data from high TB and HIV burden settings. Therefore, this study is aimed at evaluating the performance of QFT-GIT test for the diagnosis of pulmonary tuberculosis and determining the agreement of QFT-GIT test with TST in for the diagnosis of high risk populations for LTBI.

## Main text

### Methods

A cross sectional study was carried out in Saris, Wereda 6 and Wereda 7 health centers in Addis Ababa, Ethiopia. A total of 213 study participants were consecutively enrolled from September 2012 to February 2015. Participants were adult’s ≥ 18 years and were grouped into TB suspects and latent TB infection group: (1) TB suspects groups: were patients that have one or more TB specific sign and symptoms based on 2012 guidelines for clinical and programmatic management of TB, leprosy and TB/HIV in Ethiopia. TB suspects visiting the health facilities during the study period and who were voluntary for HIV testing and naïve for highly active antiretroviral therapy (HAART) and anti TB treatment were recruited consecutively. TB suspects groups were tested for smear microscopy, culture and QFT-GIT. Active TB groups are diagnosed by using smear microscopy confirmed wit culture. (2) Latent TB suspects: were healthcare workers, family care givers of peoples with active TB and voluntary counseling and testing center visitors that do not have TB specific sign and symptoms. Those individuals who were voluntary for HIV testing and not recently tested for TST (with in 2 years), participants that do not have history of allergy and naïve for HAART were included. The latent groups were subjected for TST and QFT-GIT testing. Latent TB groups are identified using TST. A structured questionnaire was prepared and socio-demographic and clinical data like sex, age, and body mass index (BMI) and TB specific sign and symptoms were collected. TST testing was done using 0.1 ml (2 TU/0.1 ml) tuberculin purified protein derivate (PPD) RT23 (Statens Serum Institute, Copenhagen, Denmark) were administrated intradermally with 1 ml graduated syringe (gauge 25 or 26) in the middle third of the left forearm. The millimeters (mm) of indurations were read within 48–72 h. Reactions were observed and interpreted following manufacturers instruction. Sputum (5 ml) (only for TB suspects) and blood (4 ml) were collected from each participant. All the samples were transported to the national HIV and TB reference laboratory of Ethiopian Public Health Institute for laboratory analysis. Sputum samples were processed using modified Petroff’s method. Sputum smears were also prepared from sediment of a processed specimen. The slides were coded, dried in air, heat fixed, and stained by Ziehl Neelsen (ZN) technique. An aliquot of 100–200 μl of sputum sample was then inoculated into two tubes of Löwenstein–Jensen (LJ) medium. Identification of *M. tuberculosis* complex from other mycobacterial species was done using TB Ag MPT64 rapid capilia test (Capilia TB-Neo, Japan). In addition to this, blood sample was added to the tube containing fluorochrome-labeled antibodies and analysed using FACS Caliber Flow cytometry analyzer for CD4 determination following the protocols of national HIV reference laboratory to determine CD4 using FACS caliber. QuantiFERON-TB In-Gold test (QFT-GIT) was performed using QFT-GIT In-tube test (Cellestis Ltd., Victoria, Australia). Briefly, 1 ml of blood was taken in each of the three tubes precoated with TB–antigen, phytohemagglutinin for the positive control or no antigen for the negative control. The cut-off point for the diagnosis was determined as per manufacturer’s instruction and the test results were interpreted using software supplied by the manufacturer (Cellestis Ltd., Victoria, Australia). Data was analyzed using STATA version 10 analytical software (Statacorp LP, USA). The performance of QFT-GIT test for the diagnosis of active pulmonary TB was compared with sputum LJ culture, the gold standard test. As there is no gold standard test for latent TB, Kappa value was also calculated to see the agreement of QFT-GIT with TST.

### Results

From the total, 136 were active pulmonary TB suspects and 66 were latent TB groups. All the TB suspects were screened for TB specific sign and symptoms and 132 (97.1%) had cough of ≥ 2 weeks and were tested by smear microscopy, culture and QFT-GIT. All the LTB groups did not have TB specific sign and symptoms (Table 1).

**Table 1:** Characteristics of the study participants

Category	Tuber suspects (N = 136)	LTB (N = 66)
Sex, N (%)		
Female	58 (42.6)	27 (50.9)
Male	78 (57.4)	26 (49.1)
Age, median in years (range)	30 (18–80)	27 (18–77)
CD4 count (Cells per µl), N (%)		
CD4 < 200	25 (18.4)	0 (%)
CD4 ≥ 200	111 (81.6)	66 (100)
BMI category (kg/m^2^), N (%)		
BMI < 18.5	41 (30.1)	7 (10.6)
18.5 < BMI < 24.99	74 (54.4)	43 (65.2)
BMI ≥ 25	21 (15.4)	16 (24.2)
BCG, N (%)		
Vaccinated	40 (29.4)	42 (63.6)
Not vaccinated	96 (70.6)	24 (36.6)
HIV status		
Positive	21 (15.4)	3 (4.5)
Negative	115 (84.6)	63 (95.5)
Smear status, N (%)		
Positive	12 (8.8%)	NA^a^
Negative	124 (91.2%)	NA
Culture, N (%)		
Positive	37 (27.2)	NA
Negative	99 (72.8)	NA
Blood with sputum, N (%)	28 (20.6)	NA
Breath shortness, N (%)	90 (66.2)	NA
Night sweats, N (%)	99 (72.8)	NA
Chest pain, N (%)	110 (80.9)	NA
Loss of weight, N (%)	91 (66.9)	NA

^a^Not applicable for testing latent tuberculosis group

From 136 suspects, QFT-GIT was positive in 75 (55.1%). Compared to the gold standard test the sensitivity, specificity, positive predictive value (PPV) and negative predictive value (NPV) of QFT-GIT for the diagnosis of active TB were 70.3% (26/37), 49.5% (49/99), 34.7% (26/75) and 83.1% (49/59) respectively. Thirty-two patients were positive by QFT-GIT and ZN microscopy, of which 21 were smear negatives but QFT-GIT positives, five were positive by both QFT-GIT and ZN and six patients were smear positive, but QFT-GIT negative (Table 2). However, of the culture confirmed patients, 26 (34.7%) were smear negatives. Compared with LJ culture, the sensitivity and specificity of ZN microscopy in this study was 29.9 and 99%, respectively. QFT-GIT were positive, negative and intermediate in 29 (50%), 27 (46.6%) and 2 (3.4%) of the females. From adults with age 18–65 years, QFT-GIT were positive in 36 (48.6%) and negative in 38 (51.4%). In those with normal BMI (between 18.5 and 24.99 kg/m^2^) QFT-GIT were positive in 36 (48.6%) and negative in 38 (51.4%). QFT-GIT results were not significantly associated with independent variables: age (p = 0.852, X^2^ = 0.32), sex (p = 0.182, X^2^ = 3.41), Bacille Calmette Guerin (BCG) vaccination (p = 0.638, X^2^ = 0.9), BMI (p = 0.163, X^2^ = 6.524), smear status (p = 0.521, X^2^ = 1.305), duration of cough (p = 0.502, X^2^ = 5.328), sputum with blood(p = 0.276, X^2^ = 2.578), breath shortness (p = 0.852, X^2^ = 3.41), night sweats (p = 0.602, X^2^ = 1.013), chest pain (p = 0.222, X^2^ = 3.0), and weight loss (p = 0.79, X^2^ = 1.094). In the HIV co infected group, QFT-GIT was positive in nine. All the nine QFT-GIT positive HIV infected peoples were smear negative. From the QFT-GIT positives, five were confirmed positive by LJ culture, but four were culture negatives. With this, the sensitivity and specificity of QFT-GIT in HIV infected individuals were 62.5 and 66.7%., respectively.

**Table 2:** Performance of QFT-GIT for diagnosis of active TB

Category	N^b^	QFN, N (%)		
		Positive, N (%)	Negative, N (%)	Intermediate, N (%)
HIV positive	21	9 (42.9)	11 (52.4)	1 (4.8)
HIV negative	115	66 (57.4)	48 (41.7)	1 (0.9)
CD4 < 200 cells/µl	25	11 (44.0)	13 (52.0)	1 (4)
CD4 ≥ 200 cells/µl	112	65 (58.0)	46 (41.1)	1 (0.9)
Smear positive	12	5 (41.7)	7 (58.3)	–^a^
Smear negative	124	70 (56.5)	52 (41.9)	2 (1.6)
Culture positive	37	26 (70.3)	10 (27.0)	1 (2.7)
Culture negative	99	49 (49.5)	49 (49.5)	1 (1.0)
BCG vaccinated	40	23 (57.5)	17 (42.5)	–^a^
BCG not vaccinated	96	52 (54.2)	42 (43.8)	2 (2.0)
Sputum with blood	28	19 (67.9)	9 (32.1)	–^a^
Breath shortness	90	52 (57.8)	38 (42.2)	–^a^
Night sweats	99	53 (53.5)	44 (44.4)	2 (2.1)
Chest pain	110	64 (58.2)	45 (40.9)	1 (0.9)
Weight loss	91	49 (53.8)	40 (44.0)	2 (2.2)

^a^Intermediate results were not found

^b^In all the percentage calculations, N was taken as denominator

From the 66 peoples with latent TB, 53 (80.3%) were TST positives. QFT-GIT were positive, negative and intermediate in 35 (53.0%), 29 (43.9%) and 2 (3%), respectively. QFT-GIT has detected two individuals that were missed by TST. However, 18 TST positive individuals were negative by QFT-GIT (Table 3). From the 42 BCG vaccinated study subjects, TST were positive in 33 (78.5%) subjects; however QFT-GIT were positive in 15 study participants. TST results were significantly associated with history of BCG vaccination (p = 0.04), but not QFT-GIT positivity (p = 2.13). From individuals with BMI (< 18.5 kg/m^2^) TST were positive and negative in 3 (42.9%) and 4 (57.1%), respectively whereas QFT-GIT were positive in 4 (57.1) and negative in 3 (42.9%). TST and QFT-GIT results were concordant in 44 study subjects, both QFT-GIT and TST were positive in 33 (50%) and both were negative in 11 (16.7%). The agreement between these tests were found to be fair with kappa = 0.316 (p = 0.001), 95% CI (1.605–1.609).

**Table 3:** Concordance of QFT-GIT with TST for the diagnosis latent TB

Tests combined^a^	Concordance, N (%)
QFT-GIT^+^ TST^+^	33 (50)
QFT-GIT^+^ TST^−^	2 (3)
QFT-GIT^−^ TST^+^	18 (27.3)
QFT-GIT^−^ TST^−^	11 (16.7)

^a^+ positive, − negative

### Discussion

The performance of QFT-GIT in this study is lower when compared to studies done in low burden developed countries [12–14]. Though findings from developing countries are heterogeneous, most of the results are similar to this study [15–18]. This was also supported with similar findings in a systemic review of studies done in low and middle income countries [19]. The difference can be explained by differences in socio-demographic and immunosuppressive diseases [20]. The sensitivity from one study done in Ethiopia was slightly lower than this study. When the cutoff value was decreased the sensitivity was improved though this affects the specificity. More studies are required to assess the discrepancy in the performance of the test in different geographical settings. In the context of HIV, the performance of QFN-GIT test was slightly decreased and relatively higher number of intermediate results was observed when compared to HIV unknown status tuberculosis patients [21]. The findings are supported by the findings from a systematic review conducted by Santin et al. [22] indicating a suboptimal sensitivity. Moreover, they also found relatively higher intermediate values in studies done in developing countries [23]. This might be due to the fact that HIV related immune-suppression affects the production of TB specific interferon gamma [24]. Moreover, the study participants are antiretroviral naïve patients in which their CD4 cells are expected to be lower than patients on treatment though the effect of antiretroviral therapy on diseasing outcome is not well known [25]. In agreement with other studies, there was no significant effect on QFT-GIT results of patients who have history of BCG vaccination. This is related with the use of highly *M. tuberculosis*-specific antigens which are not present in BCG species [26, 27].

Though cardinal diagnostic test for confirming tuberculosis suspects in developing countries is smear microscopy, we found that, the sensitivity is lower especially in HIV infected TB patients. This is due to the fact that the production of smear is compromised in patients with HIV infection [28, 29]. However QFT-GIT test were shown to detect significantly many smear negatives but culture positive patients, might help increasing the case detection rate of smear negative patients. This is more important in tuberculosis suspected HIV infected individuals, as majority of the smear negatives were HIV infected with lower CD4 counts. Though HIV-associated immune-suppression, measured by circulating CD4^+^ T-lymphocytes, negatively affects the performance of QFT-GIT, its ability to identify smear negative patients could be used as a support for microbiological tests. As peoples with LTBI serve as potential reservoirs for future infections, identifying latently infected peoples using effective tests is important. In this study higher number of positive results with TST compared to QFT-GIT was observed. However The TST results were significantly affected by BCG vaccination, as majority of the BCG vaccinated subjects were positive for TST [30]. But this was not observed in QFT-GIT tests results for the diagnosis of LTBI. The findings of this study were similar with several studies [31–33]. This signifies that QFT-GIT implementation for LTBI screening minimizing false positive or negative results [34]. The discordant results lead to a fair agreement between the two tests which is relatively lower than study done by Jang et al. but relatively good agreement compared to study done by Lee et al. [35].

In conclusion, we found relatively lower sensitivity of QFT-GIT in HIV infected than TB patients without HIV. Our data also suggested QFT-GIT test has higher sensitivity in detecting smear negative TB patients and lower specificity than AFB smear microscopy in TB patients irrespective of HIV infection. This shows the potential to use QFT alone or in combination with AFB smear for the diagnosis and or screening of active TB in smear negative patients with and without HIV infection. In addition to this, despite the significant use of QFT-GIT for diagnosis of active TB, we found fair agreement with TST for screening of LTBI. Further research on both active TB and LTBI in different groups including children and in different geographical locations of resource constrained settings is needed.

## Limitation

The small sample size in latent TB groups was difficult to see the risk factors affecting the performance of the tests in our study setting.

## Abbreviations

AFB: acid fast bacilli
BCG: Bacille Calmette Guerin
BMI: body mass index
HAART: highly active anti-retroviral therapy
HIV: human immunodeficiency virus
IGRA: interferon gamma release assays
LTBI: latent tuberculosis infection
LJ: Lowenstein Jensen
NPV: negative predictive value
PPV: positive predictive value
QFT-GIT: quantiferon TB gold in tube
TB: tuberculosis
TST: tuberculin skin test
ZN: Ziehl–Neelsen
